# Temporal Patterns of Angular Displacement of Endosomes: Insights into Motor Protein Exchange Dynamics

**DOI:** 10.1002/advs.202306849

**Published:** 2024-06-03

**Authors:** Siwoo Jin, Yongdeok Ahn, Jiseong Park, Minsoo Park, Sang‐Chul Lee, Wonhee J. Lee, Daeha Seo

**Affiliations:** ^1^ Department of Physics and Chemistry DGIST Daegu 42988 Republic of Korea; ^2^ Division of Nanotechnology, and Department of DGIST Daegu 42988 Republic of Korea

**Keywords:** 3D rotation, bio‐imaging, endosome, material transport, nanoparticle, plasmonics, serial correlation

## Abstract

The material transport system, facilitated by motor proteins, plays a vital role in maintaining a non‐equilibrium cellular state. However, understanding the temporal coordination of motor protein activity requires an advanced imaging technique capable of measuring 3D angular displacement in real‐time. In this study, a Fourier transform‐based plasmonic dark‐field microscope has been developed using anisotropic nanoparticles, enabling the prolonged and simultaneous observation of endosomal lateral and rotational motion. A sequence of discontinuous 3D angular displacements has been observed during the pause and run phases of transport. Notably, a serially correlated temporal pattern in the intermittent rotational events has been demonstrated during the tug‐of‐war mechanism, indicating Markovian switching between the exploitational and explorational modes of motor protein exchange prior to resuming movement. Alterations in transition frequency and the exploitation‐to‐exploration ratio upon dynein inhibitor treatment highlight the relationship between disrupted motor coordination and reduced endosomal transport efficiency. Collectively, these results suggest the importance of orchestrated temporal motor protein patterns for efficient cellular transport.

## Graphical abstract

A novel imaging technique allows simultaneous observation of translational and rotational motion using anisotropic nanoparticles and short‐time Fourier transform. This tool is applied to analyze 3D angular displacement of endosomes, enabling the discovery of a serially correlated temporal pattern and two types of rotational events in live cells. This approach provides insights into efficient searching behavior during intracellular material transport.

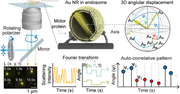

## 1 Introduction

Biological systems, such as cells, are open and complex systems that exist far from thermodynamic equilibrium.^[^ ^1^, ^2^ ^]^ They maintain a dynamic interplay with their surrounding environment by exchanging materials and energy. The orchestrated flow of substances entering and generated within the cell, along with sequential chemical reactions, perpetuates a non‐equilibrium state in both materials and energy. Nevertheless, the essence of living organisms lies in the intricate strategies employed to optimize the efficiency of these processes and uphold a state of orderliness and stability, even amidst chaos. Accordingly, elucidating the fundamental principles that organize and maintain the non‐equilibrium cellular state is a key aspect of cell biology. Cells use two strategies: i) “compartmentalization” to secure isolated spaces and promote specific biochemical reactions or prevent the dispersion of concentrated substances^[^ ^3^, ^4^ ^]^ and ii) “active transport” to deliver materials to the correct locations in a timely manner.^[^ ^5^, ^6^, ^7^ ^]^ Cells achieve the latter by transporting vesicles with motor proteins along a complex microtubule (MT) network. This transport is conducted by kinesin and dynein—opposite‐polarity MT motors—toward the (+) and (−) ends of the MTs, respectively. Notably, intracellular materials are bidirectionally transported due to the simultaneous attachment of two types of motor proteins to a single cargo, which is described as a tug‐of‐war (also known as the mechanical competition model), facilitating the forward, reverse, or stalling motion of the cargo.^[^ ^8^, ^9^ ^]^ Although this movement appears irregular, it can be suspected that the precise and consistent transportation of cargo to its destination is governed by an underlying cellular system.

**Figure 1:**
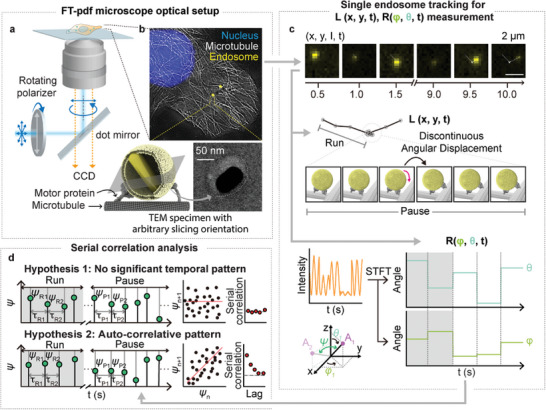
Schematic illustration for observing the temporal pattern of endosomal rotation with FT‐pdf microscopy in living cells. a) Diagram of the epi‐polarization DF microscope. A scattering signal from the plasmonic nanoparticles was observed under the illumination of rotating linearly polarized light. b) Schematics of cargo transportation by coordinated motor proteins along the MT. A DF image of an Au NR inside an endosome overlaid on the super‐resolution radial fluctuation (SRRF) image of MT (top) and a TEM image of an Au NR in an endosome (bottom) are shown. The orientation of the Au NR represents endosomal rotation since it is tightly anchored to the inner membrane. c) Schematic illustration of the simultaneous analysis of *L*(*x*, *y*, *t*) and *R*(*ψ*, *θ*, *t*). In the preliminary study, the angular displacement of endosomes was characterized by rapid and discontinuous movements. By monitoring the real‐time trajectory and scattering intensity of a single Au NR (*x*, *y*, *I*, *t*), we could extract both location information and angle change information of the endosome. For the analysis of *R*(*ψ*, *θ*, *t*), the phase and amplitude of *I* were examined using STFT. d) Illustration of the process for identifying the temporal pattern of endosomal rotation. The serial correlation in the time‐series analysis was utilized to examine the autoregressive feature of sequential endosomal rotation.

In vitro studies using motor‐bound cargos have shown that the number and combination of motor proteins play a crucial role in determining navigating behaviors, including passing, pausing, or backward movements.^[^ ^10^, ^11^ ^]^ According to the observations and the interpretations of the research conducted so far, it is necessary for motor protein exchanges, including their binding and detachment, to occur in coordination. This coordination is essential to establish the necessary motor protein pool before departure, which in turn, affects the rotational coordinates of the paused endosome.^[^ ^12^ ^]^ For instance, in neurons, the degree of endosomal rotation increases following direction reversal, providing clear evidence that motor protein exchange is influenced by historical circumstances during transport.^[^ ^13^ ^]^ Therefore, simultaneously tracking the rotational *R*(*φ*, *θ*, *t*) and translational *L*(*x*, *y*, *t*) motion of the endosome will facilitate the characterization of the endosomal motor protein exchange strategy in response to stimuli encountered during transportation (**Figure** **1**a–c).

From our initial investigations, we also observed that the angular displacements of an individual endosome exhibit rapid, discontinuous, and stochastic behavior due to the motor exchanges taking place between the vesicle and MT (Figure S1, Supporting Information).^[^ ^12^, ^14^ ^]^ Consequently, cells likely employ a programmed strategy to manage motor protein exchange sequences and achieve a statistically higher expected reward (increased probability of reaching the destination) rather than leaving their fates to random chance, which would result in a lower expected reward. To investigate this and directly observe the *R*(*φ*, *θ*, *t*), earlier investigators have devised techniques to monitor the angular displacement of endosomes through the utilization of various combinations of microscopic techniques with anisotropic probes (e.g., fluorescence microscopy with Janus nanoparticles,^[^ ^14^ ^]^ multichannel dark‐field [DF] microscopy,^[^ ^13^ ^]^ planar illumination microscopy, and differential interference contrast [DIC] microscopy with gold nanorods [Au NRs]).^[^ ^15^, ^16^, ^17^ ^]^ Notably, Au NRs, in conjunction with optical polarization splitting to observe rotation in 3D space, are excellent tools for studying the behavior of endosomes in living cells over long periods of time without disruption.

In this study, we present a straightforward and practical microscopic methodology using DF microscopy with Au NRs (Figure 1c). Instead of decomposing the 3D orientation through optical installation, we employed Fourier transformation (FT) to distinguish between azimuthal (*φ*) and polar angles (*θ*). This was accomplished by applying FT to the DF signal while it was subjected to rotating polarization conditions. By subjecting the DF signal to FT analysis, we were able to decompose it into its constituent angular orientation components. Particularly important in investigating the stochastic properties of motor protein pool exchange during transport is the calculation of the serial correlation coefficient (SCC) from the trajectories of *R*(*φ*, *θ*, *t*) (Figure 1d). Through this investigation, our objective is to analyze and clarify the temporal dependencies and correlations in successive observations of motor protein exchange events. This analysis is crucial for understanding the complex mechanisms that drive the stochastic behavior exhibited by motor protein transport dynamics.

Here, we developed an FT‐based plasmonic DF microscope (FT‐pdf) to monitor the real‐time 3D rotation of cargo and analyze the serial causality of endosomal traces. The FT‐pdf microscopy approach introduced in this work measures the dynamic 3D orientation of anisotropic imaging probes. Utilizing this approach, we successfully elucidated the intricate translational and rotational dynamics of cargo containing Au NR. Compared to previous methods that demonstrated success (e.g., polarization splitting or defocused imaging), this approach offers advantages in terms of ease of use, localization, and signal‐to‐noise ratio, as only one polarizer needs to be placed in the path of light.

## 2 Results and Discussion

### 2.1 FT‐Pdf Microscope for Assessing Rotational Displacements in 3D Space

For FT‐pdf, the Au NRs were used as anisotropic probes and visualized using epi‐type DF microscopy. Linearly polarized light was directed onto the Au NRs through an aluminum‐patterned dot mirror to achieve partial illumination. Simultaneously, a motorized rotation stage continuously rotated the polarized light for FT (Figure 1a). Au NRs provide an excellent system for studying intracellular transport owing to their polarization angle dependency, stable optical properties without blinking or bleaching, and simple surface chemistry through Au–thiol bonds.^[^ ^18^, ^19^, ^20^, ^21^ ^]^ A key principle in determining the 3D orientation of Au NRs using FT‐pdf microscopy is the simultaneous measurement of the azimuthal (*φ*) and polar (*θ*) angle‐dependent signals (Figure 1c). In epi‐polarization DF microscopy, when Au NRs are observed under rotating linearly polarized light (*φ_pol_*), the oscillating scattering intensity (*I_NR_*) caused by angle‐dependent localized surface plasmon resonance can be observed. Given that the *I_NR_* peaks when *φ_pol_* is parallel to *φ*, and its trough when *φ_pol_* is perpendicular to *φ*, the value of *φ* can be determined based on the relative phase of the *I_NR_*. Furthermore, the amplitude of the *I_NR_* wave, which is affected by the orthogonal projection length, can be used to determine the *θ* value (Figure S2, Supporting Information).

Validation experiments were conducted to ensure the accuracy of Au NR localization and orientation using DF microscopy. We first obtained a nonpolarized DF image and a sequence of DF stacks for the Au NRs fixed on the glass while changing the *φ_pol_* to analyze (*x*, *y*) and *φ* coordinates. The position and orientation of the Au NRs were identified by localizing the center and measuring the maximum intensity, respectively. The resulting coordinates (*x*, *y*, *φ*) of each NR were well‐matched with the scanning electron microscopic image for the same region (Figure S3, Supporting Information). Regarding *θ*, since the empirical verification of the dependence on *θ* is influenced by typical microscope excitation changes, we conducted a finite‐difference time‐domain (FDTD) calculation to examine the *I_NR_* when the Au NR was vertically rotated under linearly polarized light. Similarly, the calculated intensity of *θ* followed the sine function, which was linearly proportional to the vertical projection length of Au NR, indicating that *θ* was visualized in the amplitude of the signal when the *I_NR_* was observed with a rotating polarizer (Figure S2b, Supporting Information). Therefore, *R*(*φ*, *θ*, *t*) and *L*(*x*, *y*, *t*) could be reconstructed if phase and amplitude information from the oscillating *I_NR_* was obtained.

To achieve this, we computed the FT separately on local sections of the signal; that is, short‐time FT (STFT) was used to determine the phase and amplitude content of the corresponding polarizer frequency component as it changed over time. Given the uniform distribution of motor protein docking adaptors on cargo,^[^ ^22^, ^23^ ^]^ the occasional transition of the motor protein pool in a single endosome exhibits no angular bias, suggesting a simultaneous abrupt change in both *φ* and *θ*. To detect the common transition juncture of bivariate time‐series data, *F*(*t*) = [*φ*(*t*), *θ*(*t*)], we adopted a change‐point detection algorithm.^[^ ^24^, ^25^ ^]^ After testing our analysis pipeline using synthetic data, we verified a strategy to identify points where two variables change simultaneously, preventing false detection by spike noise and sensibly detecting the rotational event that can be missed when a single variable is applied (Figure S2c–g, Figure S4, and Movie S1, Supporting Information). These results confirmed the feasibility of FT‐pdf microscopy to investigate the sequence of motor protein exchange during endosomal transport.

### 2.2 Lateral and Rotational Motion of a Single Endosome in a Living Cell

To apply FT‐pdf microscopy to track the lateral and rotational displacement of endosomes in live‐cell lamellipodia, we internalized Au NRs through clathrin‐mediated endocytosis as a model system.^[^ ^26^, ^27^ ^]^ Clathrin‐mediated endosomes play a crucial role in the transportation of molecules, including receptors, into cells. This process is essential for regulating signal transduction pathways, nutrient uptake, and the internalization of pathogens. To achieve this, first, the surface of Au NRs was sequentially modified via carboxylic acid functionalization (─COO^−^),^[^ ^28^ ^]^ and transferrin (Tf^+^) coating with electrostatic interactions (Figure S5, Supporting Information).^[^ ^29^ ^]^ The resulting Tf‐coated Au NRs (Au NR@Tf) were specifically attached to the cell without lateral movement and were internalized while maintaining a tightly bound state (Figure S6, Supporting Information). In addition, transmission electron microscopy (TEM) revealed that individual Au NRs were internalized in the endosome and tightly enclosed by the lipid membrane (Figure 1b). Thus, we confirmed that the *I_NR_* from a single endosome should have a sinusoidal form for polarization without interference by plasmonic coupling or the superposition of several signals. In addition, due to the intact emplacement of the Au NR through multiple binding events inside the endosome, the 3D rotation of the endosomes can be represented by the spherical coordinates of the Au NR over a long period. Note that, in control experiments using Au NR‐COO^−^, minimal intracellular uptake of Au NRs was observed (Figure S5g,h, Supporting Information). Some nonspecifically introduced nanoparticles exhibited rapid and random rotations driven by Brownian motion within the confined space of the endosome (Figure S6g, Supporting Information). Through comparative experiments on internalization, which focused on surface chemistry and TEM analysis, it was observed that a majority of Au NRs are internalized via Tf mediation. Furthermore, these AU NRs were found to be tightly localized within the endosomal membrane.

**Figure 2:**
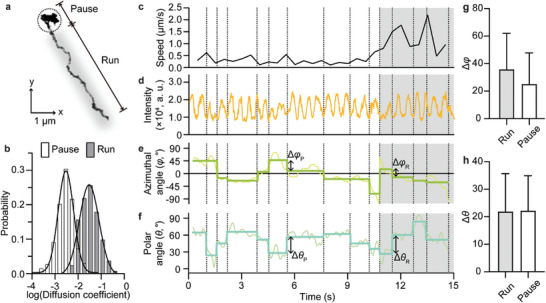
Measurement of rotational moments of endosomes during pause and run in living cells. a) Trajectory of an endocytic vesicle in a living cell. The internalized Au NR@Tf was observed under linearly polarized light with a rotating speed of 360° s^−1^. b) Probability histogram of instantaneous diffusion coefficient derived from the trajectory. Run and pause states were discriminated by a 10^−2.3^ µm^2^ s^−1^ cut‐off. c−f) Analysis of the endosomal trajectory represented in (a) that involves instantaneous speed (c), *I_NR_* (d), *φ* (e), and *θ* (f). The grey area indicates the run state, and the white area represents the pause state. Instantaneous speed was calculated every 500 ms. As the lower baseline of scattering intensity remains constant, the Au NR was observed with negligible z‐axis movement in the flat lamellipodial MT. Discontinuous 3D rotation of endosome was analyzed using STFT; the rotational moments are marked by dotted lines along the vertical axis. g,h) Mean and standard deviation (error bar) of *∆φ* (g) and *∆θ* (h) during run or pause (*n* = 385 and 878 for the rotational event at run and pause, respectively). The rotational events at the moment of transition from run to pause and vice versa were excluded.

In our system, the vesicle containing an Au NR traveled a long distance (>5 µm) with a velocity of 0.74 ± 0.42 µm s^−1^ (**Figure** **2a**) and halted at the crossroads of the microtubules; the converse case was not consistently observed (Figure S7, Supporting Information). These results are consistent with those of previous studies,^[^ ^30^, ^31^, ^32^, ^33^ ^]^ indicating that FT‐pdf microscopy enables long‐term observations of endosomal movement without perturbing transport. Through the mean‐squared displacement analysis, the endosomal trajectory can be classified into two states based on the instantaneous diffusion coefficient *D* (two distinct distributions, *D* < 10^−2.3^ µm^2^ s^−1^ and *D* > 10^−2.3^ µm^2^ s^−1^) (Figure 2a–c). Considering the anomalous exponent at each state (*α*, *α* = 1: normal diffusion; *α* > 1: super‐diffusion; *α* < 1: confined diffusion),^[^ ^34^, ^35^ ^]^ the calculated *α* (*α* = 0.57 ± 0.19 or *α* = 1.33 ± 0.25) (Figure S8, Supporting Information) suggests that endosomal movements can be classified as either “pause” or “run” states. Moving forward, subscripts will be employed to represent each state, such as *D_pause_*, *α_run_*, and *Δφ_run_*.

In parallel, we analyzed the oscillating *I_NR_* to extract real‐time rotational coordinates (*φ* and *θ*) of the Au NR inside the endosome (Figure 2d). As in the demonstration using synthetic data, we characterized the abrupt change in endosomal rotation in living cells using STFT and the change‐point detection algorithm (Figure 2e,f; Movie S2, Supporting Information). The lower envelopes (baseline) of the obtained *I_NR_* waves remained constant. This indicates that plasmonic Au NR signals were acquired from endosomes in the lamellipodia, which is the flat region of the cell; therefore, there was a negligible change in the z‐axis and no significant interference with the signal. The alteration of *φ* (Δ*φ*) had a higher value in the run state than in the paused state (Δ*φ_pause_* = 25.1 ± 22.5° and Δ*φ_run_* = 35.8 ± 26.1°), while the transition of *θ* (Δ*θ*) had a similar value in both states (Δ*θ_pause_* = 22.2 ± 12.7° and Δ*θ_run_* = 21.9 ± 13.7°) (Figure 2g,h). This difference in the rotational motion of the endosome under the run and paused states suggests the possibility that the rotation is primarily physically caused by the sterically hindered environment (two MTs crossing each other). This implies that the exchange of motors is governed by different stochastic processes depending on the navigating situation. The cells likely regulate endosome navigating strategies by regulating the expression of MTs and their density and shape.

Note that: (1) A singular angular displacement serves as a comprehensive measurement that temporally averages various single‐step reactions. This includes processes such as the binding, detachment, and exchange of diverse adapter proteins, encompassing motor proteins, intermediates between them, and their dynamic equilibrium. (2) Our analysis assumes that the highest and lowest *I_NR_* values recorded during the presence of a single endosome in the lamellipodia (which lasts ≈3 min and consists of 1 × 10^3^ frames, with angle changes occurring 1 × 10^2^ times) correspond to the horizontal and perpendicular directions of polarization, respectively. Although this assumption aligns with the statistical distribution of the highest/lowest values of *I_NR_* in the 3D space filled by the Au NRs, it is still unknown if the recorded maximum and minimum values of each individual endosome perfectly align in a perpendicular and horizontal manner to polarization. Put simply, it is important to recognize that this assumption has limitations when considering the numbers, which could lead to an overestimation of all observed angular shifts. However, the distribution of angular alterations and their temporal patterns, as will be further examined, may possess significant implications on their own.

### 2.3 Characterizing the Temporal Pattern of Endosomal Rotation during Tug‐of‐War

**Figure 3:**
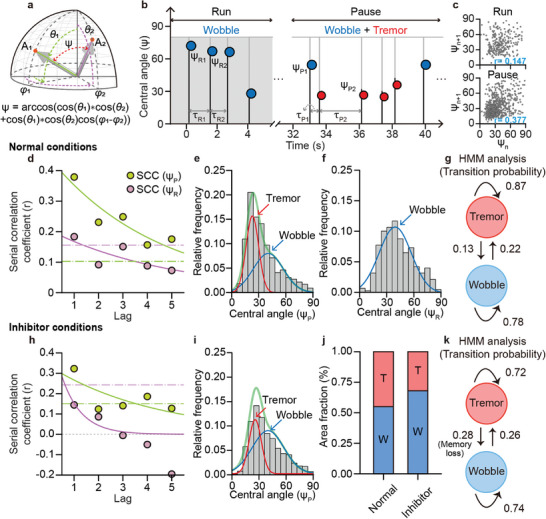
Increased Markovian iteration frequency between two rotational modes at pause by a motor protein inhibitor. a) Schematic illustration showing the *ψ* before and after endosomal rotation. b) Sequence of *ψ* and inter‐rotational duration (*τ*) during the pause and run. c) SCC of *ψ_P_* and *ψ_R_* under normal conditions. Statistical cut‐offs were determined by calculating the top 0.1% value in the empirical null distributions. The *ψ_P_* exhibited a significant positive correlation for lag 5, whereas *ψ_R_* showed a negligible correlation. The SCC was fitted with the exponential decay function. c) Serial correlation between *ψ_N+1_* and *ψ_N_*. For lag 1, the pause state has a significant r value. d,e) Probability histogram for *ψ_P_* (d) and *ψ_R_* (e) fitted with a single and a mixture of two Gaussian functions, respectively. The Gaussian component with a large variation is denoted as the wobble, and the other with a small variation as the tremor. f) Markovian transition diagram of rotational modes at pause under normal conditions. g) SCC of *ψ_P_* and *ψ_R_* under inhibitor conditions. Both *ψ_P_* and *ψ_R_* showed no significant correlation. h) Probability histogram for the *ψ_P_* (*n* = 1066 for the rotational event at pause). i) Comparison of the fraction of rotational modes at pause (the relative ratio of wobble:tremor was 0.45:0.55 under normal conditions and 0.32:0.68 under inhibitor conditions). j) Markovian transition diagram of rotational modes at pause under the inhibitor condition. The cut‐offs for statistical significance were determined by using the value corresponding to a p‐value of 0.001 in the empirical null distribution.

Based on the time points at which the rotation took place and the gyroscopic coordinates before and after rotation, we calculated the sequence of inter‐rotational duration (*τ*) and great circular angle changes (*ψ*) of endosomes (**Figure** **3**a,b). To elucidate the temporal pattern of the sequential stochastic rotation of endosomes, we applied the SCC and the hidden Markov model (HMM). The SCC entails examining the correlation between a signal and a delayed version of itself at various time intervals. This enables us to observe how one event affects the subsequent events in a sequence of random events; it is applied in several fields, including single‐molecule chemistry^[^ ^36^, ^37^, ^38^ ^]^ and computational neuroscience,^[^ ^39^, ^40^ ^]^ to examine the autoregressive features of sequential events. The SCC plot showed that serial *ψ* and *τ* had significant positive SCCs for lag 5 and lag 2 in the paused state, whereas *ψ* and *τ* sequences during the run state had less significant SCC values (Figure 3c,d; Figure S9–S11, Supporting Information). These results indicated that the replacement of motors on the endosome during the tug‐of‐war was regulated by a temporally controlled stochastic process, whereas the rotation during the run exhibited a non‐sequential dependency.

To further assess the model for rotational temporal patterns, we focused on *ψ*, which exhibited a higher tendency toward the SCC, particularly for the differences between the distribution of *ψ* with and without the sequential pattern (i.e., pause and run, respectively). The Gaussian mixture model decomposition revealed that the distribution of *ψ* in the pause state (*ψ_P_*) consists of the sum of two Gaussian distributions (hereafter, wobble and tremor) with different mean *µ* values (*µ_wobble_* = 39.1 ± 17.9°, *µ_tremor_* = 22.3 ± 6.6°), whereas the *ψ* in the run state (*ψ_R_*) shows exclusively one distribution with the higher average value among the two (wobble only) (Figure 3e,f). This implies that (1) there are two distinct modes for the change in the motor protein pool of a single endosome, and (2) tremors are suppressed during the run state; however, the mechanism underlying this process remains to be elucidated.

To validate the SCC results, we used the HMM for analysis,^[^ ^41^ ^]^ which is a commonly employed machine learning algorithm for analyzing sequences of correlated events and their transition probabilities from different sources. In our study, we applied the HMM to the sequence of rotational angles observed during pauses. Our analysis revealed a state transition diagram that showed a significantly higher probability of recurrence (e.g., ≈0.7 when wobble → wobble, and tremor → tremor) compared to the probability of transition (e.g., ≈0.3 when wobble → tremor, and tremor → wobble) (Figure 3g). Alternately, once the endosome switches from its motor protein exchange mode to another during the tug‐of‐war, it may be sustained through subsequent rotational events. Therefore, the sporadic Markovian transition between the hidden motor protein exchange modes (wobble and tremor) is the causal factor of a positive SCC of the rotational angle at pause.

In this experiment, we observed two rotational modes and tried to interpret their nature. The wobble mode occurred in both pause and run states. Considering the common characteristics of endosomal transport (e.g., change in cargo velocity, overcoming microtubule intersection),^[^ ^13^, ^15^, ^32^ ^]^ endosomal rotation is derived through the attachment and detachment of occupied motors on the surface of the endosome and MTs. This process can result in a wide range of random rotations depending on the distribution of occupied motors; thus, we consider this case a wobble. However, tremors only appeared in the paused state. In previous reconstituted microtubule experiments, researchers revealed that dynein‐coated beads halt and anchor at intersections under high densities, whereas single motors show various behaviors such as passing, dissociation, and switching.^[^ ^10^, ^11^ ^]^ These discoveries led to the suggestion that the intersections of microtubules play a role as tethering sites, and this tethering capability can be tailored by modulating the motor stoichiometry within cells.^[^ ^10^ ^]^ Due to the balance between competitive motors at pause, such a tug‐of‐war probably maintains the rotational tension tightly. Under this condition, adding nearby motors or losing the possessed motors (updating the motor population) leads to a slight flinching of the endosome, which corresponds to a tremor. This understanding implies that endosome pausing forms an appropriate motor combination according to the current surrounding environment.

Although our observations demonstrate molecular biological phenomena, such as the movement of endosomes and exchange of associated proteins, they do not provide a definitive answer to intracellular material transport. However, the temporal patterns we observed, which involve several tremors or wobbles following an initial tremor or wobble, allow for several interpretations. First, it suggests that motor exchange within endosomes is not random from a statistical perspective. Second, localized and transient cellular environments consistently favor one form of protein exchange. Third, although speculative, these temporal dynamics resemble finding strategies such as Levy walks or multi‐armed bandits. Consequently, we cautiously pose the question: “Could cells possess sophisticated molecular biological strategies to efficiently deliver small cargoes to specific locations?” We invite readers and researchers alike to contemplate this proposition.

### 2.4 Effects of Dynapyrazole‐A on the Temporal Pattern of Endosomal Rotation

Based on our proposed scenario, the temporal characteristics of endosomal rotation are affected by the number of occupied motor proteins. For example, an endosome with a high density of motor proteins is more likely to prefer the transition to tremors (including recurrent events) because rigid motor–MT interactions interrupt the relatively large gyroscopic orientation changes. To test this hypothesis, we performed an SCC analysis of the rotational sequence under the dynapyrazole‐A treatment condition. Dynapyrazole‐A is an inhibitor of the MT‐stimulated ATPase activity of dynein, leading to an overall reduction in motor protein levels in the cell. We confirmed the effect of the inhibitor by observing a 40% decrease in vesicle velocity (0.44 ± 0.35 µm s^−1^) compared with that in the control condition (Figure S12, Supporting Information), consistent with previous literature reports.^[^ ^42^ ^]^ We also found that the SCC of *ψ_P_* was significantly reduced by dynapyrazole‐A, and subsequent HMM analysis revealed that the inhibitor treatment increased the transition probability from tremor to wobble by approximately two‐fold (Figure 3h−k). This result is reasonable, as decreasing the binding of dynein to MTs reduces the physical anchoring of endosomes, thereby increasing the likelihood of wobbling.

However, we observed that the wobble‐to‐wobble recurrence rate remained similar regardless of dynapyrazole‐A treatment. This result suggests that absolute motor protein levels are required for proper wobble‐to‐wobble self‐transition. The non‐significant difference in the recurrence rate of the wobble before and after dynapyrazole‐A treatment can be attributed to a decrease in dynein levels, which lowers the recurrence rate of wobble as well as the wobble‐to‐tremor transition (Figure 3k). Together, these factors shifted the steady distribution of endosomal rotation toward the wobble state (Figure 3i,j) and a decrease in the SCC (Figure 3h). Although these findings may not fully explain the molecular mechanism underlying the recurrent manifestation of wobbles or tremors, they provide insights into the temporal pattern of active transport, governed by a combination of surrounding motor proteins.

**Figure 4:**
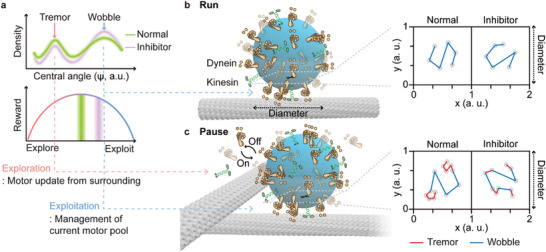
Interpretation of two endosome rotational modes using the exploration and exploitation framework. a) Illustration of the relative ratio of tremor to wobble under normal and inhibitor conditions. To describe the transition of rotational modes, the concept of exploration–exploitation was introduced; exploration implies expanding new information, whereas exploitation refers to utilizing known knowledge. Only a well‐adjusted tradeoff between them can yield optimal results. b,c) Schematic illustration of endosomal rotation during run and pause. When mapping the 3D rotation of an endosome in the projection plane, the wobble and tremor are represented as long and short displacements, respectively. Since the wobble is observed in all cases and the tremor only appears at pause, the wobble can be characterized as exploitation that utilizes the current motor pool and the tremor as exploration that updates the motors around the endosome. Compared with the normal conditions, treatment with dynapyrazole‐A increased the exploitation/exploration ratio, suggesting that motor inhibition limits the adaptability of new information and lowers transport efficiency.

Our analysis revealed the presence of two rotational modes during material transport toward its destination. This result shares similarities with the concept of exploitation and exploration, which are commonly employed in decision‐making and foraging behaviors (**Figure** **4**).^[^ ^43^, ^44^, ^45^, ^46^ ^]^ In such scenarios, an agent must decide between exploiting the current choice, assuming it is optimal with the given current information, and exploring alternative options to gather more information and potentially achieve better outcomes. A straightforward approach to achieving optimal results involves gathering sufficient information and maintaining the best policy. Therefore, a strategy that considers the tradeoff between exploitation and exploration, along with their recursive nature, is crucial for achieving functional performance. In our endosomal rotation model, tremors correspond to exploration because the cellular system is programmed to gather information regarding the concentration of surrounding motor proteins. Conversely, a wobble corresponds to exploitation, as it evaluates whether a combination of proteins is suitable for transport. We hypothesize that exploitation and exploration recursiveness, quantified with the SCC, is an optimized transport efficiency that may be dependent on the cell type or environmental situation. The disruption of the balance between the wobble and tremor caused by treatment with dynapyrazole‐A supports this proposal. Furthermore, an increase in the exploitation portion under dynapyrazole‐A‐treated conditions compensates for the decrease in transport efficiency resulting from frequent exploration and exploitation. This interpretation provides a deeper understanding of the efficient transport strategy underlying the control of the exploitation–exploration transition frequency.

## 3 Conclusion

In summary, we developed a novel imaging technique to simultaneously observe translational and rotational motion with an anisotropic nanoparticle, which shows potential for versatile usage due to the accurate measurement in localization and orientation. Using FT‐pdf microscopy, we investigated the endosomal transport over a long time, confirmed discontinuous 3D angular displacement, and found a serially correlated temporal pattern of the rotational events in live cells. We revealed that the two types of rotational motion during tug‐of‐war under normal conditions exhibited an altered transition frequency and ratio under the dynein inhibitor condition. The data presented in this study provide insights into cargo dynamics with the collective behavior of occupied motor proteins, which enable efficient material transport. We hope this rotational study offers a more extensive and profound understanding of how the programmed cell efficiently regulates motor exchange depending on surrounding environments.

## 4 Experimental Section

### Materials

For general experiments, acetic acid (99.7%, Sigma‐Aldrich, St. Louis, MO, USA), acetone (99%, Alfa Aesar, Ward Hill, MA, USA), agarose powder (low EEO, Sigma‐Aldrich), Alconox detergent (Sigma‐Aldrich), dimethyl sulfoxide (DMSO, 99%, Sigma‐Aldrich), ethanol (EtOH, 96%, Alfa Aesar), sodium hydroxide beads (NaOH, ≥ 97%, Sigma‐Aldrich), and Tris/Borate/EDTA buffer (TBE, Sigma‐Aldrich) were purchased and used without further purification. Deionized water (DI water, 18.2 MΩ) was used throughout the experiment.

For the synthesis of Au NR@Tf, ascorbic acid (99%, Sigma‐Aldrich), 5‐bromosalicylic acid (90%, Sigma‐Aldrich), hexadecyltrimethylammonium bromide (CTAB, 95%, Sigma‐Aldrich), holo‐transferrin human (Tf, ≥ 97%, Sigma‐Aldrich, UniProt No. P02787), 2‐(N‐morpholino)ethanesulfonic acid buffer (MES, Thermo Fisher Scientific, Waltham, MA, USA), sodium borohydride (NaBH_4_, 98%, Sigma‐Aldrich), silver nitrate (AgNO_3_, 99%, Sigma‐Aldrich), tetrachloroaurate trihydrate (99.9%, HAuCl_4_, Sigma‐Aldrich), and HS‐(CH_2_)_11_‐(OCH_2_CH_2_)_6_‐OCH_2_CO_2_H (HSC_11_EG_6_CO_2_H, ProChimia) were used.

For cell culture and transfection, collagen I (3.6 mg mL^−1^, rat tail, Santa Cruz Biotechnology, Dallas, TX, USA), Dulbecco's Modified Eagle Medium (DMEM, high‐glucose, Biowest, Nuaillé, France), fetal bovine serum (FBS, Biowest), pAcGFP1‐Tubulin plasmid (PT3836‐5, Clontech, Takara Bio, Kusatsu, Japan), 10× phosphate‐buffered saline (PBS, Sigma‐Aldrich), 100× penicillin–streptomycin solution (Biowest), 0.25% trypsin‐EDTA (Biowest), and U2OS cells (Korean Cell Line Bank, KCLB No. 30 096) were used.

For the observation of Au NRs in endosomes using an electron microscopy, an Embed‐812 kit (Electron Microscopy Sciences, Hatfield, PA, USA), ethyl alcohol (99.5%, Sigma‐Aldrich), glutaraldehyde (50% in H_2_O, Sigma‐Aldrich), osmium tetroxide (99.8%, Sigma‐Aldrich), propylene oxide (99%, Sigma‐Aldrich), formalin solution (10%, neutral buffered, Sigma‐Aldrich), sodium cacodylate (98%, Sigma‐Aldrich), and uranyl acetate (98.0–102.0%, Electron Microscopy Sciences) were used.

### Optical Microscopy

DF, DIC, and total internal reflection fluorescence (TIRF) microscopy were performed with an inverted microscope (ECLIPSE Ti2‐E, Nikon, Tokyo, Japan) equipped with a motorized stage (Ti2‐S‐SE‐E), a perfect focus system (Ti2‐N‐ND‐P), a stage‐top‐incubator controlling temperature and CO_2_‐concentration (UNO‐T‐H‐PREMIXED, Okolab, Pozzouli, Italy), and an electron‐multiplying charge‐coupled device (iXon Ultra 897, Andor, Oxford Instruments, Abingdon, UK). A mercury lamp (C‐HGFIE Intensilight, Nikon), LED lamp (TI2‐D‐LHLED, Nikon), and laser (LU‐N4 Laser Unit; wavelength, 488 nm; Nikon) were used as light sources for DF, DIC, and TIRF imaging, respectively. The DF images were acquired using an aluminum patterned dot mirror installed in a cube box. The polarized DF images were obtained under a linear polarizer (LPVISE50‐A with N‐BK7 windows, Thorlabs, Newton, NJ, USA) equipped in a motorized rotation stage (DDR25/M, Thorlabs) combined with a DC Servo Motor Controller (KBD101, Thorlabs). SRRF images were acquired by analyzing 100 frames of stack images using the SRRF‐stream algorithm. All DIC images were captured using a 20× lens (0.75 NA, CFI Plan Apochromat, Nikon) and a DIC slider (MBH76220, Nikon). DF, TIRF, and SRRF images were captured with 60× and 100× objective lenses (Nikon, 1.49 NA, oil immersion, CFI Apochromat TIRF).

### Synthesis of Au NR@Tf

A three‐step sequential synthetic strategy was used to prepare Au NR@Tf:

1. Au NRs. The Au NRs were synthesized following published methods, with slight modifications.^[^ ^47^ ^]^ The procedures were categorized into the preparation of seed and growth solutions. To prepare the seed solution, 0.5 mm HAuCl_4_ (5 mL) was mixed with 0.2 m CTAB solution (5 mL) in a 20 mL vial. Next, fresh 6.0 mm NaBH_4_ (1 mL) solution was added into the Au‐CTAB solution under vigorous stirring for 2 min. The seed solution color was immediately altered to yellowish brown and matured for 30 min before use. To make the growth solution, CTAB (4.5 g) and 5‐bromosalicylic acid (0.55 g) were prepared in a 250 mL Erlenmeyer flask and dissolved in DI water (125 mL) by sonication for 3 h. The solution was cooled to 30 °C. Next, 4.0 mm AgNO_3_ solution (6 mL) was added to the mixture; this mixture was left undisturbed for 15 min. Then, 1.0 mm HAuCl_4_ (125 mL) solution was added to the reaction solution and mixed at 400 rpm. After 15 min, 64 mm ascorbic acid (1 mL) was injected; then, the solution was stirred until it became colorless. Next, the aged seed solution (20 µL) was added to the growth solution. The reaction mixture was stirred for 5 min and left undisturbed for 12 h. The products were purified by centrifugation twice at 6,000 rpm for 10 min and redispersed in 10 mm CTAB solution (extinction coefficient: 7.35 × 10^9^ m ^−1^cm^−1^ at 641 nm).
2. Carboxylic Acid‐Functionalized Au NRs (Au NR‐CO_2_H). Au NR‐CO_2_H was synthesized following published methods, with slight modifications.^[^ ^28^ ^]^ To remove excess CTAB, 100 µL of the as‐synthesized Au NRs (10 nm) were washed twice with DI water by centrifugation at 6000 rpm for 5 min. The pellet was mixed with 10 mm HSC_11_EG_6_CO_2_H (50 µL) in 1.5 mL epi‐tube. After shaking for 24 h, the reaction product was purified by centrifugation at 6000 rpm for 10 min and stored at 4 °C.
3. Au NR@Tf. The final product was synthesized following published methods, with slight modifications.^[^ ^48^ ^]^ Next, the as‐synthesized Au NRs‐CO_2_H (10 nm, 10 µL), 1 mm holo‐Tf solution (10 µL), and MES buffer (10 µL, pH 5.7) were mixed in a 1.5 mL epi‐tube. After shaking overnight, the reaction product was purified by centrifugation at 6,000 rpm for 10 min. The reaction product was redispersed in DMEM and immediately used.

### Characterization

The Au NRs were characterized by field‐emission TEM (HF‐3300, Hitachi, Tokyo, Japan) under 300 kV conditions. For sample preparation, a TEM grid (Formvar/Carbon on 300 mesh, Ted Pella, Redding, CA, USA) was treated with O_2_ plasma using an advanced plasma cleaner (950 M, Gatan, AMETEK, Berwyn, PA, USA). Subsequently, Au NR solution (10 µL) was loaded onto the TEM grid and allowed to dry. The average size was measured using ImageJ/Fiji software by counting the particles.^[^ ^49^ ^]^ The endocytic vesicle containing an Au NR was visualized by bio‐TEM (Tecnai G2 F20 TWIN TMP, FEI, Hillsboro, OR, USA) at 80 kV. To validate the positional and angular accuracy of Au NR in the DF image, the particles loaded onto glass coverslips (grid‐50, ibidi, Gräfelfing, Germany) were observed using field‐emission scanning electron microscopy (SU8020, Hitachi) at a voltage of 10 kV and a current of 10 µA. Optical absorption spectra were recorded using a UV‐vis spectrophotometer (Cary8454, Agilent Technologies, Santa Clara, CA, USA). The net surface charges of the Au NRs suspended in DI water were measured by Zetasizer nanoseries (MAL1160456, Malvern Panalytical, Malvern, UK). Electrophoresis was performed using a Mini‐Sub Cell GT Cell (Bio‐Rad, Hercules, CA, USA) with 1.5% agarose gel in TBE buffer (pH 7.6) at 150 V for 10 min.

### Cell Fixation for TEM

To observe endosomes containing an Au NR via TEM without cell deformation, a slightly modified published method was used.^[^ ^50^ ^]^ Briefly, live cells were washed with sodium cacodylate buffer (0.2 m, pH 7.4) at 37 °C. The cells were fixed at room temperature for 20 min with a mixture of glutaraldehyde (2.5% v/v) and formaldehyde (2.0% v/v) in the buffer. They were then left undisturbed at 4 °C overnight and subsequently washed with the buffer. Next, the cells were incubated with an osmium tetroxide second fixative solution (1% v/v) for 40 min and washed with buffer and water. For sample dehydration, a gradient series of EtOH solutions in DI water (30%, 50%, 70%, 80%, 90%, 95%, and 100%) was used. After removing the EtOH, the samples were treated with propylene oxide and left for 2 h. As part of the embedding process, the samples were incubated in graded mixtures of propylene oxide and resin (3:1, 1:1, and 1:3) for 2 h each. Then, the samples were incubated in pure resin for 2 h after removing the excess resin. The samples were solidified by incubation in an oven at 68 °C for 48 h. The samples were cooled down using liquid N_2_ to remove the glass from the resin. Next, the embedded cells were sectioned into ultrathin layers (≈100 nm) using a cryogenic ultrathin microtome (EM UC7, Leica, Wetzlar, Germany), and the sectioned films were collected on TEM grids (Formvar on 200 mesh, Cu, Ted Pella). For negative staining, the grids were incubated in filtered uranyl acetate (1%) for 20 min in the absence of light and then rinsed with water. After drying, the samples were ready for observation using a bio‐TEM.

### Localization of Particle Position and Orientation

The fixed Au NRs on glass were observed via DF microscopy. The background signals were subtracted using ImageJ/Fiji. To mark the center of each particle, the point spread function was fitted with an integrated Gaussian curve using the ThunderSTORM algorithm (Imagej/Fiji plugin).^[^ ^49^, ^51^ ^]^ To measure the orientation, the DF image sequences were acquired with a rotating polarizer, and the scattering intensity time trace was fitted with a sine function. The reconstructed position and orientation of the particle were verified by matching with SEM images captured at the same location. To accurately determine the positions of samples for both scanning electron microscopy and optical microscopy, a glass bottom dish was utilized equipped with a grid pattern (Mattek, P35G‐1.5‐14‐C‐GRID) enabling precise coordinate identification.

### Cell Culture and Transfection

U2OS cells were cultured in DMEM containing 10% FBS and 1× penicillin–streptomycin condition and passaged every 3–4 d in T25 flasks (Corning, Corning, NY). The cells were maintained under 5% CO_2_ conditions at 37 °C in a humidified incubator (Model C 170, Binder, Tuttlingen, Germany). For the fluorescence imaging of microtubules, the U2OS cells were transfected with a pAcGFP1‐Tubulin plasmid (PT3836‐5, Clontech) using a neon transfection system (MPK5000, Thermo Fisher Scientific), following the manufacturer's instructions. The transfected cells were treated in media containing G418 (500 µg mL^−1^) for 2 weeks, and the stable cell line was maintained. The purchased U2OS cell line was used at passage 18, and subsequent passages were maintained under 30.

### Imaging of Au NR@Tf in a Live Cell

For microscopic observations, the appropriate cells (70% confluency) were plated on collagen I‐coated 35 mm glass‐bottom dishes (P35G‐0‐10‐C, MatTek, Ashland, MA, USA). Next, the cells were treated with 50 µL of the as‐synthesized Au NR@Tf (100 pm), followed by incubation for 20 min at 37 °C. The unbound nanoparticles were washed with excess DMEM containing 10% FBS. Live‐cell images were observed at the lamellipodium to minimize intensity fluctuation derived from z‐axis movement. The images were captured at 85 Hz with no delay for FT‐pdf.

### Dynein Motor Inhibition via Dynapyrazole‐A

For the dynein inhibition experiments,^[^ ^42^ ^]^ the plated cells were incubated in DMEM containing 0.5% FBS for 24 h. The Au NR@Tf labeled cells were treated with 10 µm inhibitor media solution (2 mL, 0.1% DMSO) and incubated for 1 h. Then, the sample was immediately observed via polarized DF microscopy.

### Single Particle Tracking and Evaluating Diffusive Characteristics

The TrackMate plugin for ImageJ/Fiji^[^ ^52^ ^]^ was used to obtain the position and intensity of a single Au NR in an endosome. The diffusion coefficient *D* and anomalous exponent *α* for each observed sequence of jump distance *r* were evaluated by mean‐squared displacement analysis (the first four points of lag time *τ* were used for linear fitting) based on Equation (1).

(1) $$\langle r^{2}\left(\tau \right)\rangle =4D\tau^{\alpha }$$

To classify lateral movement of the endosome (run or pause), a cut‐off standard for anomalous exponents was established by the numerical simulation of Brownian particles with external drift or trapped by a confined domain.

### STFT and Finding Abrupt Changes in Both Rotational Coordinates

FT is a mathematical tool that breaks an original signal into a combination of sinusoidal base functions expressed using Euler's formula. Discrete FT is a special type of FT that is used to determine the finite sequence of a signal with equal sampling time. Any sequence of discrete signals can be expressed in Fourier series, as shown in Equation (2):

(2) $$x_{n}=\frac{1}{N}\sum_{k\;=\;0}^{N-1}X_{k}\cdot e^{\frac{j2\pi kn}{N}},\;n\in \left[0,\;N-1\right],\;j=\sqrt{-1}$$

Coefficients for the series can be expressed as follows:

(3) $$X_{k}=\sum_{n=0}^{N-1}x_{n}\cdot e^{-\frac{j2\pi kn}{N}},k\in \left[0,\;N-1\right]$$

Therefore, the complex number *X_k_* (which is easily computed using the MATLAB built‐in fast Fourier transform (FFT) algorithm^[^ ^53^ ^]^ indicates the amplitude and phase of basis oscillators with a certain frequency:

(4) $$X_{k}=Re\left(X_{k}\right)+Im\left(X_{k}\right)j=\left|X_{k}\right|\cdot e^{j∠X_{k}}$$

(5) $$\begin{array}{ccc} & & \left(basis\;function\;of\;Fourier\;series\right)=X_{k}\cdot e^{\frac{j2\pi kn}{N}}=\left|X_{k}\right|\cdot \\ & & e^{j\left(\frac{2\pi kn}{N}+∠X_{k}\right)}:basis\;wave\;with\left\{\begin{array}{c} frequency\;:f_{s}\frac{k}{N}\\ amplitude\;:\;\left|X_{k}\right|\\ phase\;:\;∠X_{k} \end{array}\right. \end{array}$$

*f_s_:sampling frequency*

STFT is one of the variants of Fourier‐related transform. It is used to determine the frequency and phase components of local sections of a signal as they change over time.

(6) $$X_{k}\left[m\right]=\sum_{n=0}^{N_{w}-1}x_{n,m}\cdot e^{-\frac{j2\pi kn}{N_{w}}}$$

(7) $$x_{n,m}=x_{n+m}\cdot w\left[n\right],n\in \left[0,\;N_{w}-1\right],m\in \left[0,\;N-\left(N_{w}-1\right)\right]$$

*w[n]:window function for masking x_n_*

The uniform window function was used for convenience in the calculation, as shown in Equation (8):

(8) $$w\left[n\right]=\left\{\begin{array}{cc} 1 & n\in \left[0,\;N_{w}-1\right]\\ 0 & else \end{array}\right.$$

STFT was conducted with a window length corresponding to one cycle of the oscillating signal (here, *N_w_* = 43‐time points = 0.5 s). For every sliding window, the spectrum amplitude and relative phase were calculated with the same FFT algorithm.

(9) $$X_{k}\left[m\right]=Re\left(X_{k}\left[m\right]\right)+Im\left(X_{k}\left[m\right]\right)j\;=\left|X_{k}\left[m\right]\right|\cdot e^{j∠X_{k}\left[m\right]}$$

To obtain the amplitude and phase of the wave with the period of *N_w_* (the oscillating signal caused by the rotating polarizer), we solely used the Fourier component corresponding to *k* = 1.

Then, those quantities were converted into rotational coordinates (azimuthal angle *φ* and polar angle *θ*) using Equation (10) and (11):

(10) $$\varphi =\frac{∠X_{k}\left[m\right]}{2}\left(angular\;speed\;of\;rotating\;polarizer\;=\;360^{\circ }\;s^{--1}\right)$$

(11) $$\theta =\sin^{-1}\left(\frac{\left|X_{k}\left[m\right]\right|-\min\left(\left|X_{k}\left[m\right]\right|\right)}{\max\left(\left|X_{k}\left[m\right]\right|\right)-\min\left(\left|X_{k}\left[m\right]\right|\right)}\right)$$

Note that Equation (11) for calculating the polar angle has been adopted from the literature^[^ ^15^ ^]^ based on the assumption that if a sufficient number of rotations is observed, the entire range of the polar angle can be covered with high probability. To fulfill this sufficient number of rotations, the trace of the endosome was observed over a long period of time (> 3 min, which encompasses more than 100 rotations), but the polar angle may have been overestimated compared to the actual degree due to the limitations in assumptions.

Before the change‐point detection analysis, outlier removal was performed via robust locally weighted scatterplot smoothing^[^ ^54^, ^55^ ^]^ because the sudden moment of rotation causes spiky noise during the measurement of *θ* and *φ*. The smoothing procedure follows these steps:

1. Calculate the residuals from the quadratic polynomial Savitzky–Golay filtering.^[^ ^56^ ^]^
2. Compute the robust weights for each data point.
(12) $$w_{i}=\left\{\begin{array}{cc} {\left(1-{\left(r_{i}/6MAD\right)}^{2}\right)}^{2} & \left|r\right|<6MAD\\ 0 & \left|r\right|\geq 6MAD \end{array}\right.$$

*MAD* = *median*(|*r*|)*where* *r*: *residuals*;  *r_i_*: *residuals* *of* *i^th^* *data* *point*

1. Smooth the data again using the robust weights.
2. Repeat steps (3) and (4) five times.

During this process, the spanning window length for Savitzky–Golay smoothing was set as half of the STFT window length. The whole process was conducted using MATLAB's built‐in data‐smoothing function.

Next, the change‐point detection algorithm was employed, as shown in Equation (13).^[^ ^24^, ^25^ ^]^ Briefly, the change‐point detection algorithm aims to minimize the total residual error (*J*) from the best horizontal level for each section with a penalized contrast (*βK*):

(13) $$J\left(K\right)=\sum_{r=0}^{K-1}\left[\sum_{i=k_{r}}^{k_{r+1}-1}\left[\Delta \left(x_{i};mean\left(x_{k_{r}},\text{…},x_{k_{r+1}-1}\right)\right)\right]\right]+\beta K$$

*K*: *number* *of* *segments*;  β: *penalty* *to* *prevent* *overfitting*

*k*_0_ = 0;  *k_K_* = *N*;  *k_r_*: *index* *for* *k^th^* *changepoint*

Δ(*x*; μ): *Euclidean* *distance* *between* *x* *and* μ

To perform the minimization, MATLAB's built‐in exhaustive algorithm based on dynamics programming was used. In this study, *β* was set as 20. We also set another parameter, minimum length (ML) between change points, to be marginally longer than one cycle (ML = 50 > *N_w_* = 43), based on the presumption that the polarizer was sufficiently quick in relation to the rotational frequency of the endosome. Next, the average values of *θ* and *φ* in each segment (discontinuous state notation), which was split by the change‐point detection algorithm, were used as the spherical coordinates of endosomal transient rotation. Note that *θ* was renormalized using this discontinuous state of *θ* under the same assumption for *θ*, as shown in Equation (11).^[^ ^15^ ^]^

All calculations for STFT, outlier filtering, and change‐point detection were conducted based on the built‐in functions in MATLAB (*fft*, *smooth*, and *findchangepts*, respectively). Raw data and codes for demonstrating the whole analysis process are provided at GitHub (https://github.com/DaehaSeo/Serial‐Correlation‐of‐Angular‐Displacement‐of‐an‐Endosome‐in‐Living‐Cell).

### HMM Analysis

HMM was used to analyze the transition rate between two Gaussian‐distributed sources of endosomal rotation *ψ* (great circular angle change). The state transition matrix of HMM was calculated by HaMMy software.^[^ ^57^ ^]^

### SCC Calculation

SCC was calculated using Equation (14):

(14) $$SCC_{k}=\frac{\sum_{t=k+1}^{T}\left(y_{t}-\bar{y}\right)\left(y_{t-k}-\bar{y}\right)}{\sum_{t=1}^{T}{\left(y_{t}-\bar{y}\right)}^{2}}$$

*where* *t*: *time* *index*;  *k*: *time* *lag*;  *T*: *length* *of* *sequential* *data*; $y_{t}:value\;of\;sequential\;data\;at\;time\;index\;t;\;\bar{y}:mean\;of\;sequential\;data$

To determine the statistical significance of SCC, 10 000+ synthetic random sequences of rotational angles were obtained by permutating the order of original rotational sequences. Then, the SCC was calculated for every random sequence to obtain an empirical null distribution. The top 0.1% (which corresponds to a one‐tail p‐value = 0.001) of SCC values were used as cut‐offs for statistical significance.

## Conflict of Interest

The authors declare no conflict of interest.

## Supporting information

Supporting Information

Supplemental Movie 1

Supplemental Movie 2

## Data Availability

The data that support the findings of this study are available from the corresponding author upon reasonable request.
