# Supplementary material for: Temporal Patterns of Angular Displacement of Endosomes: Insights into Motor Protein Exchange Dynamics
Source: Adv Sci (Weinh). 2024 Jun 3;11(29):2306849. doi: 10.1002/advs.202306849 (PMC11304332; doi:10.1002/advs.202306849)
Supplement: Supplementary file 1 — Supporting Information [file ADVS-11-2306849-s001.pdf]

ADVANCED SCIENCE
Open Access

Supporting Information

for *Adv. Sci.*, DOI 10.1002/advs.202306849

Temporal Patterns of Angular Displacement of Endosomes: Insights into Motor Protein Exchange Dynamics

Siwoo Jin, Yongdeok Ahn, Jiseong Park, Minsoo Park, Sang-Chul Lee, Wonhee J. Lee and Daeha Seo**

Supporting Information

Serial Correlation Analysis of Angular Endosome Displacement in Living Cells: Balancing Angular Exploration and Exploitation during Tug-of-War

Siwoo Jin, Yongdeok Ahn, Jiseong Park, Wonhee J. Lee, and Daeha Seo**

Supporting Figures

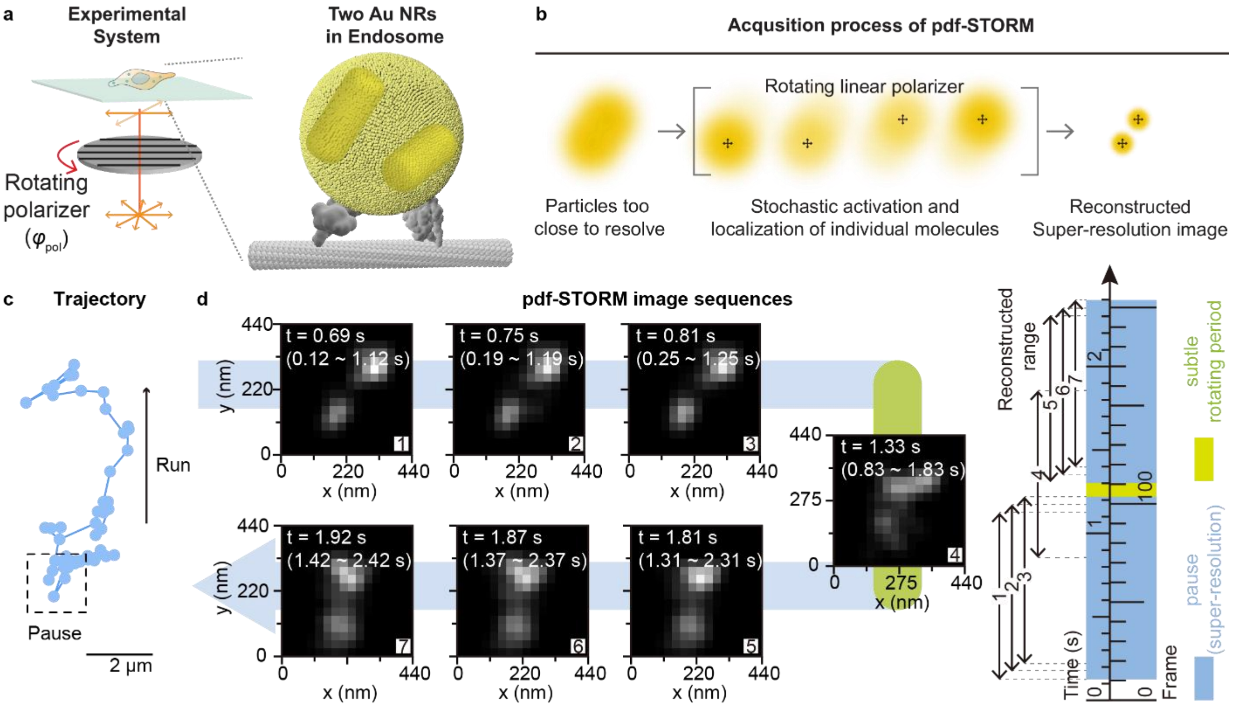

Figure S1. Analyzing the discontinuous rotation of an endocytic vesicle using pdf-STORM (plasmonic dark-field-STochastic Optical Reconstruction Microscopy). a) Experimental scheme for pdf-STORM. The two Au NRs within the endosome were observed with a rotating polarizer. b) Acquisition process of pdf-STORM. The polarized DF image sequences were captured at a rate of 85 Hz with a rotating polarizer at 360° s^{-1} and reconstructed using 85 frames. The pdf-STORM images were obtained by localizing the point spread function of individual particles and reconstructing the image sequences, which resolves two particles within the diffraction limit. The rotation of endosomes for polar and azimuthal angles was marked with changes in interparticle distance and rotation with the same interparticle distance, respectively. c, d) Trajectory of an endosome containing two Au NRs. The pdf-STORM image sequences were acquired by sequentially sliding the reconstruction range. Notably, when the center of the reconstruction range approached a discontinuous and rapidly shifting region, four well-separated spots became simultaneously observable (image 4). By comparing pdf-STORM images before and after rotation (images 1–3 and 5–7, respectively), significant alterations were observed, enabling the measurement of subtle rotating periods.

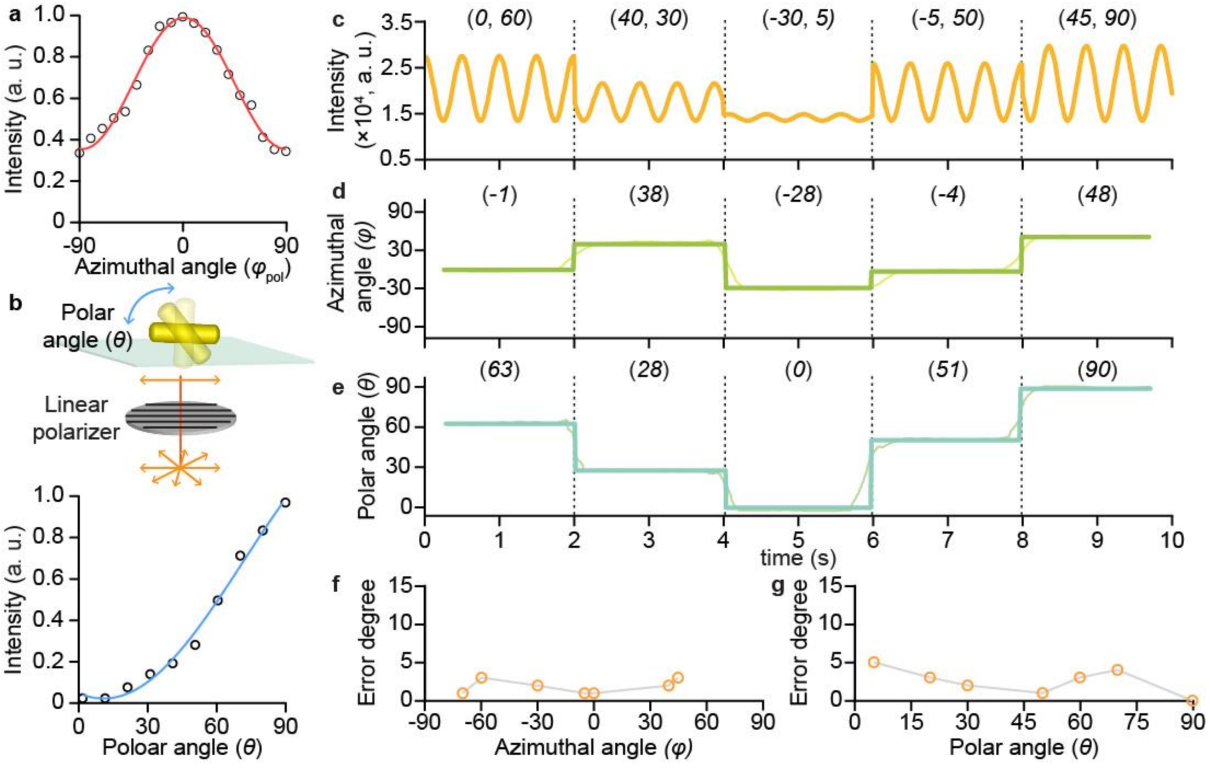

Figure S2. FT-pdf microscopy using the polarization-dependent optical characteristics of Au NRs. a) Scattering intensity of Au NRs measured with rotating linearly polarized light. The φ-dependent intensity of Au NRs is fitted with a sine function. b) Calculated FDTD intensity of Au NRs tilted by θ under linearly polarized light. The obtained θ-dependent FDTD intensity is fitted with a sine function. Considering the polarization-dependent optical property, the changes in the φ and θ of Au NRs were visualized with the alteration of relative phase and amplitude, respectively. c) Numerical experiments conducted to simulate the 3D rotation of Au NRs. d, e) Reconstruction of the φ (d) and θ (e) represented in (c) using the STFT algorithm. The moments of rotational event are marked by a dotted line along the vertical axis. f, g) Estimation accuracy of both φ (f) and θ (g). The error is less than 5° in both cases. a. u. stands for arbitrary unit. Numbers in (c), (d), and (e) indicate intended rotational coordinate (φ, θ) and reconstructed φ and θ.

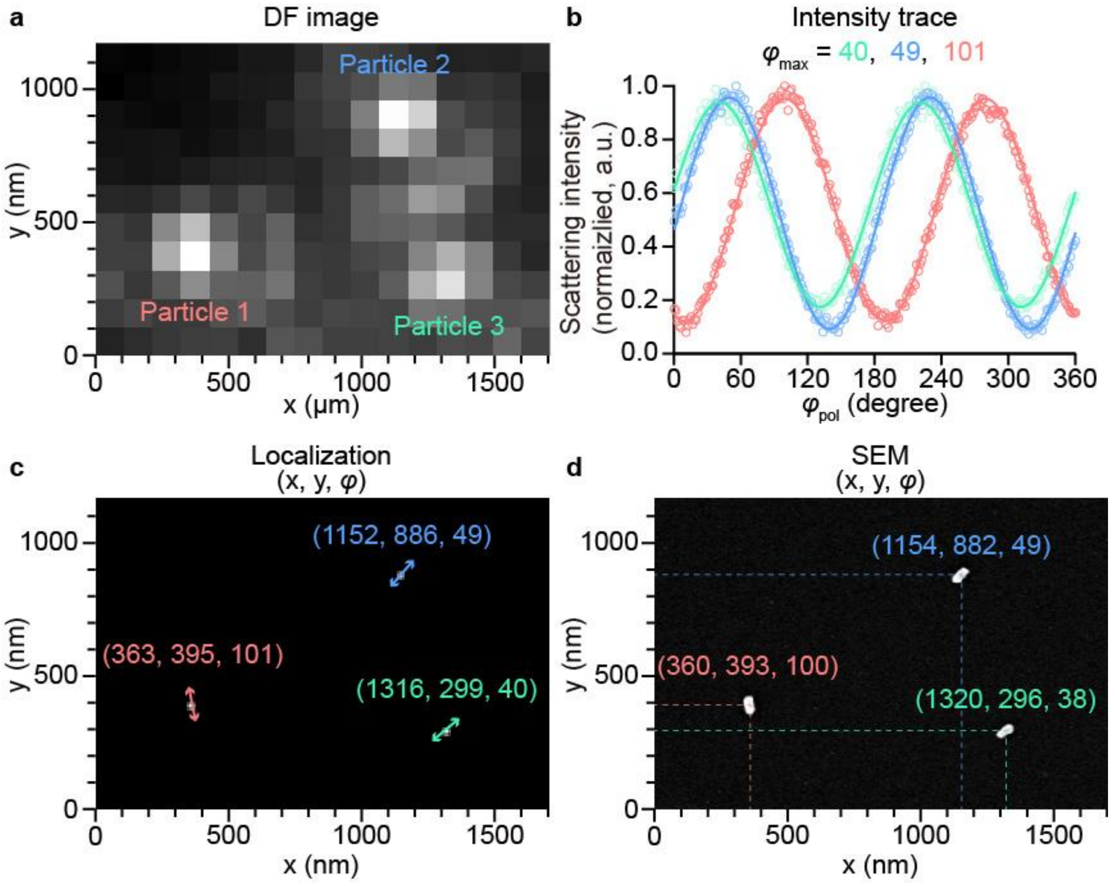

Figure S3. Verifying the positional and angular information of particles, obtained by FT-pdf, using scanning electron microscopy. a) DF image of Au NRs fixed on the glass surface without a polarizer. b) Scattering intensity of Au NRs measured by rotating linearly polarized light. Each intensity trace was fitted with a sine function. c) Sub-pixel localization of Au NRs using an integrated Gaussian function method. The positional and angular information is overlaid on the image. d) Scanning electron microscopy image captured at the same location where the DF image was obtained. Compared to those of scanning electron microscopy, the positional and angular error of FT-pdf are less than 5 nm and 3°, respectively.

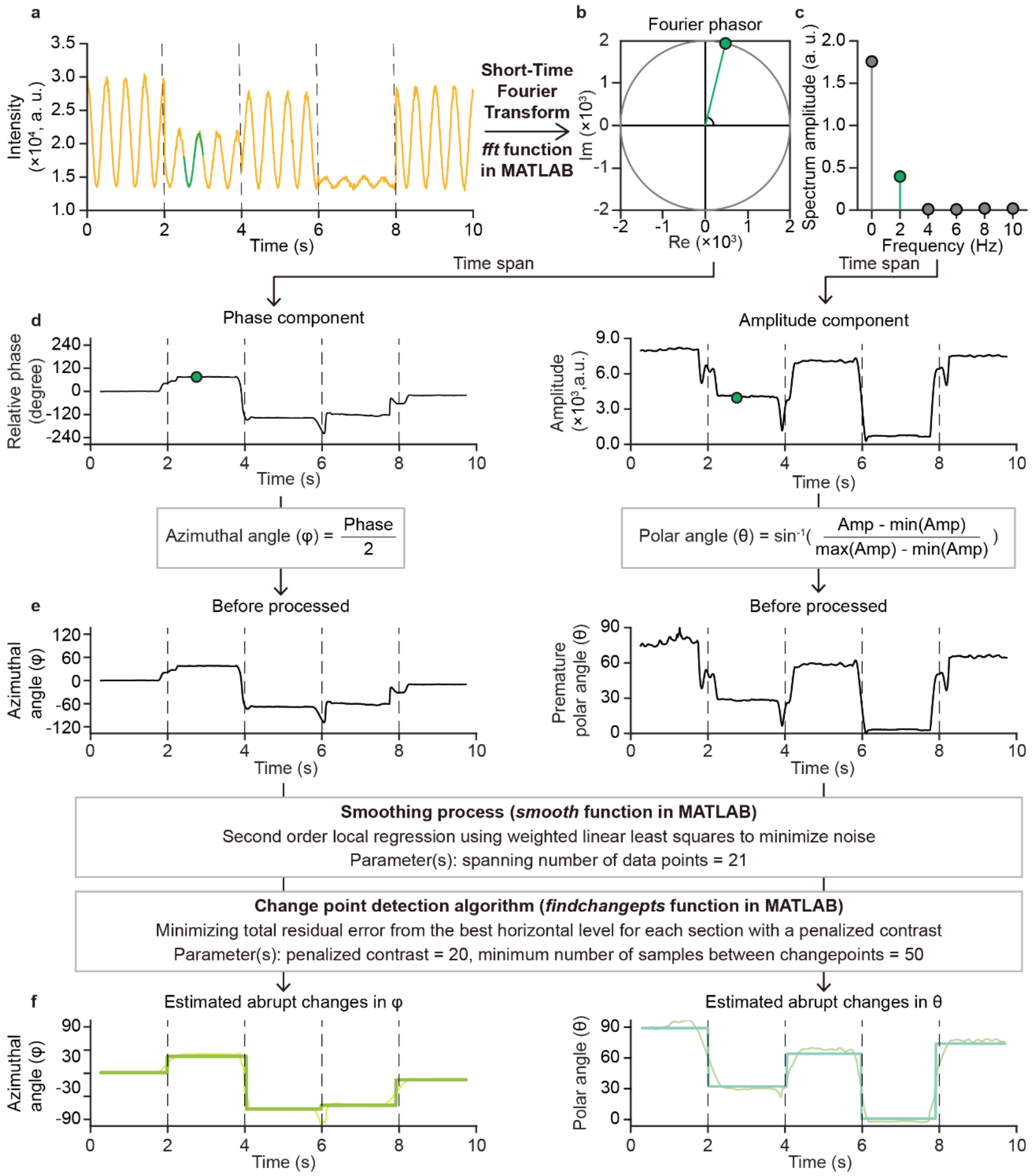

Figure S4. Detailed pipeline of data processing to identify the timing and degree of Au NR 3D rotation. a) Simulated time trace of scattering intensity postulating the abrupt change in spherical coordinates of Au NRs under rotating linearly polarized light with a rotation rate of 360° s^{-1}. b, c) Outcome of STFT. STFT was performed with a time window of 0.5 s, which corresponds to one cycle of polarizer rotation (green line in (a)). b) Phasor diagram representing the phase component of a single oscillation cycle. c) Amplitude spectrum representing the oscillation amplitude of a single oscillation cycle. d) Time trace of relative phase and amplitude, which were calculated for each with sliding window. e) Time trace of φ and θ before processing. The φ was calculated by dividing the phase in half. The θ was acquired by taking the inverse sine function for the change in amplitude. f) Time trace of processed φ and θ. The outlier was eliminated by robust local regression since spiky noise appeared around the moment of abrupt rotation. The change-point detection algorithm was applied to detect simultaneous changes in φ and θ at a specific time. A full description of signal processing is provided in the Experimental Section.

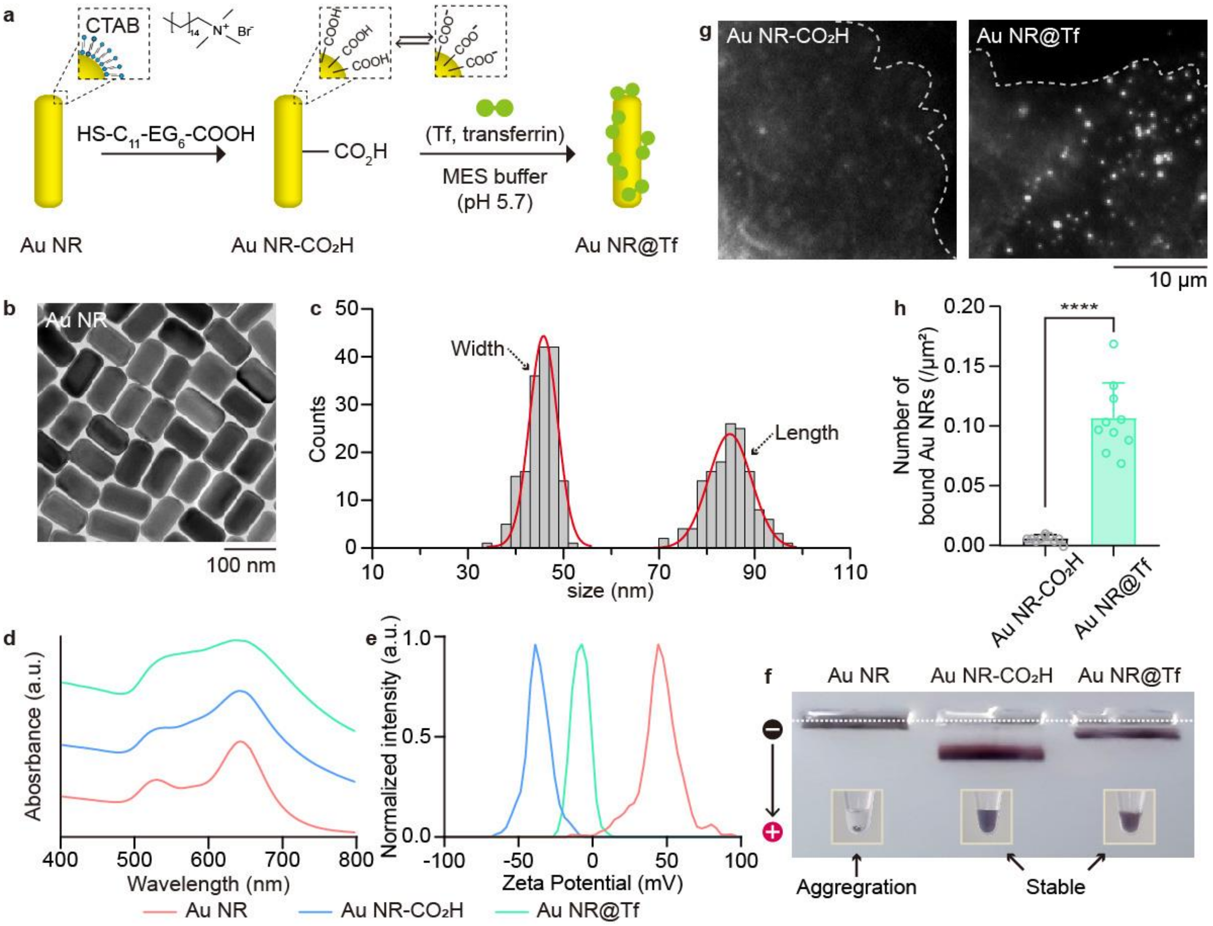

Figure S5. Synthesis and characterization of Au NR@Tf. a) Schematic illustration displaying the procedure for Au NR@Tf preparation. The Au NRs were synthesized via a seed-mediated growth method, followed by surface modification with carboxylic acid-functionalized PEG. The Tf molecules were coated onto the Au NRs through electrostatic interactions to induce clathrin-mediated endocytosis and to maintain a tightly bound position inside the endosome. b, c) TEM image (b) and size distribution histogram (c) of Au NRs. The measured width and length of Au NRs were 84 ± 5 nm and 45 ± 3 nm, respectively. d−f) UV-vis spectrum (d), zeta potential distribution (e), and gel electrophoresis (f) of surface-modified Au NRs. Absorbance peaks for the modified Au NRs are positioned at 641−642 nm. The average zeta potentials of bare Au NRs, Au NR-CO_2H, and Au NR@Tf in DI water were 44.5, −38.9, and −7.2 mV, respectively. The bare Au NRs were aggregated at the starting line of the gel, and the others showed fast mobility depending on the overall surface net charge, demonstrating the colloidal stability and successful conjugation of Tf molecules onto the Au NRs. g) DF image showing the specific labeling of Au NR@Tf on the cell surface. h) Au NR binding rate on the cell surface. The Au NR@Tf exhibited approximately a 20-fold higher cell attachment efficiency than the bare Au NRs. A parametric Student’s *t* test was used for the statistical analyses; ****p-value < 0.0001.

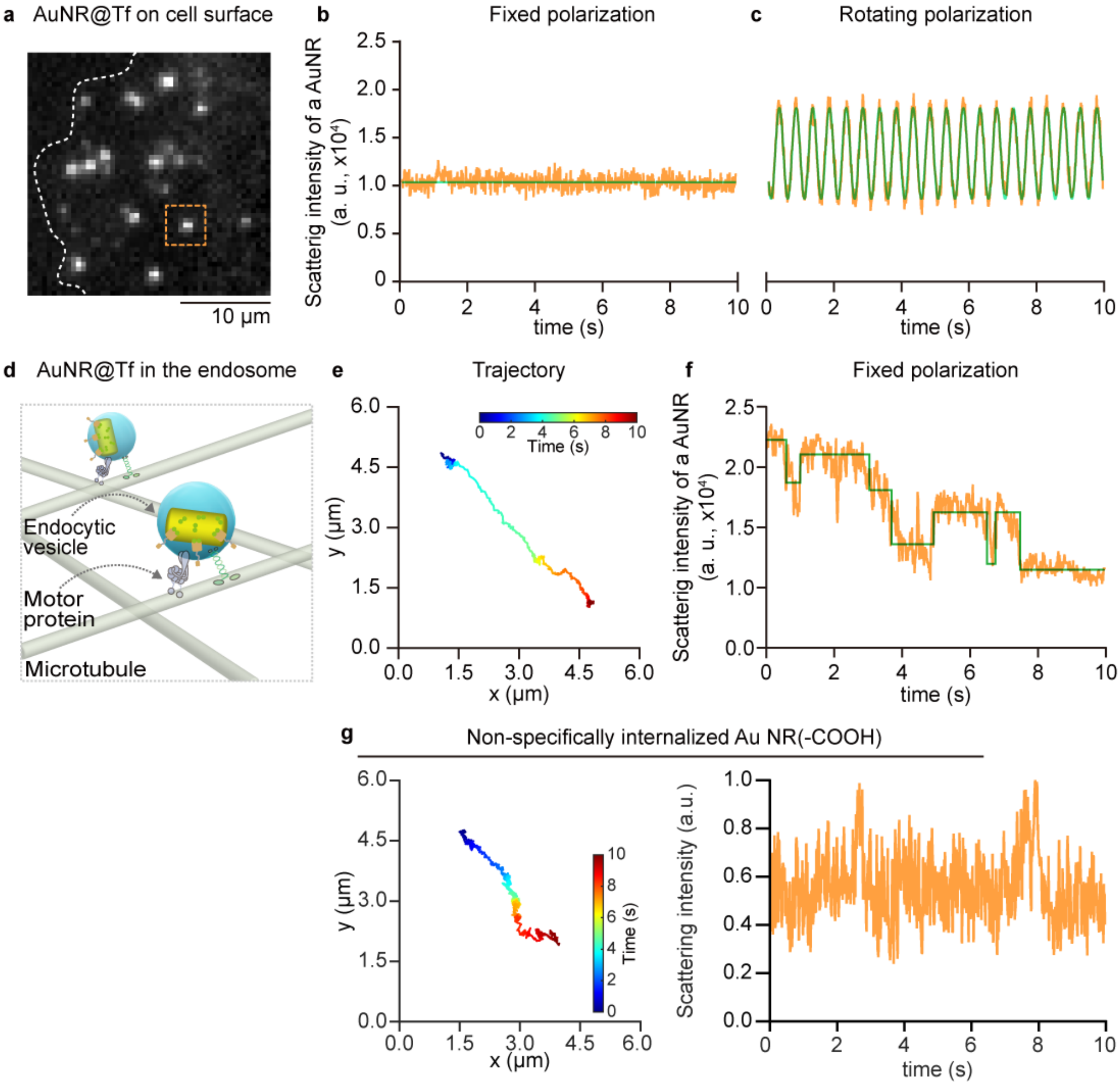

Figure S6. Intensity time trace showing immobilization of Au NR@Tf on the cell surface and inside an endosome. a) DF image of Au NR@Tf tightly attached on the surface of the lamellipodium. b, c) Time trace of scattering intensity observed under fixed (b) and rotating (c) linearly polarized light after the adhesion of the Au NR@Tf on cell surface (marked using the orange dotted box in (a)). Linear and sine functions were used to fit the intensity graphs. This suggests that the Au NR was rendered immobile by the multiple Tf molecules on it being anchored to the plasma membrane. d) Schematic illustration of an Au NR inside an endosome. e) Trajectory of an internalized endosome for 10 s. f) Time trace of the scattering intensity of the Au NR represented in (e) under fixed polarizer conditions. The discontinuous transition of the scattering intensity indicates that the rotation of the Au NR is not caused by Brownian motion but by the intermittent exchange event of motor proteins occupied by the endosome. (g) Trajectory and scattering intensity time trace of non-specifically internalized Au NR(-COOH) as a control experiment.

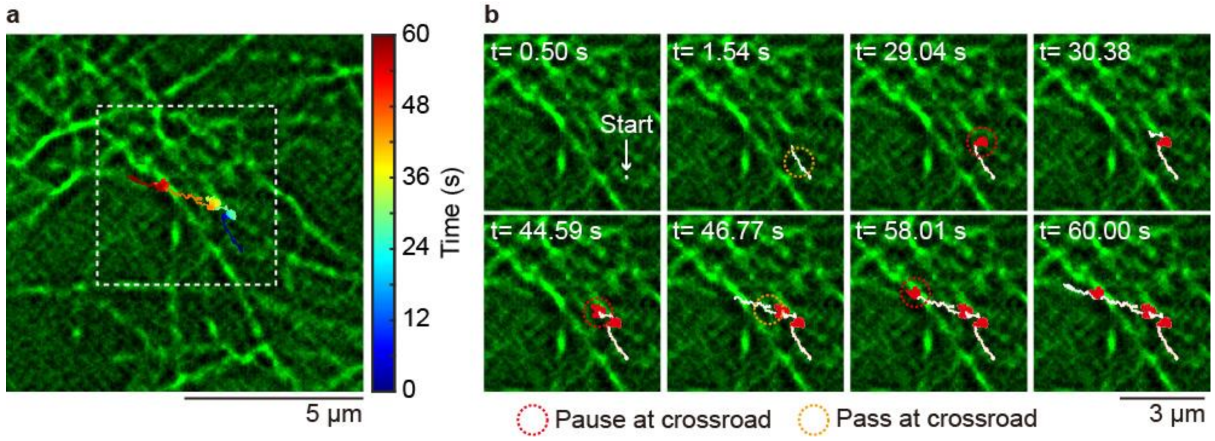

Figure S7. Monitoring of endosomal movement at the MT crossroad. a) Trajectory of an endosome moving along an MT. Lateral movement of the endosome over time was color-coded and overlaid onto a SRRF image of MTs. b) Multiple frames extracted from the DF movie of endosomes, depicting the identical region outlined with a white inset in (a). The time points when pause (red line) and run (white line) were switched are specified. The red and orange dotted circles indicate the pause and pass events at the MT crossroad, respectively. Note that the pause state occurred only at the MT crossroad; the converse is not always true.

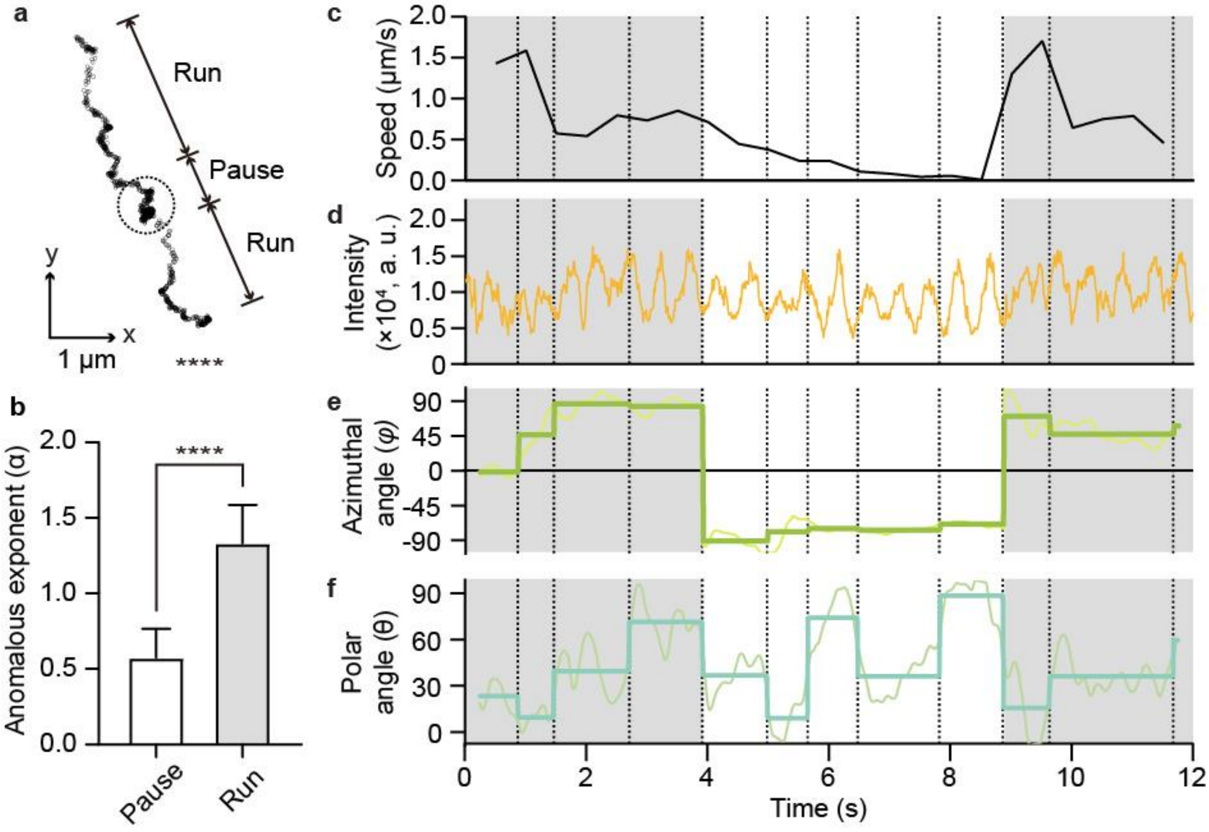

Figure S8. Observation of 3D endosomal rotation during active transport. a) Trajectory of an Au NR@Tf in an endocytic vesicle. The particle was visualized under linearly rotating polarized light with a rotation rate of 360° s^{-1}. b) Comparison of α for pause and run states. c−f) Descriptions of translational and rotational motion analysis for the track represented in (a), which show speed (c), scattering intensity (d), φ (e), and θ (f). The white and gray boxes represent pause and run states, respectively. The intensity trace of Au NR was analyzed by FT-pdf, and the relative phase and amplitude were converted to φ and θ, respectively. Intermittent rotational events are denoted by dotted lines along the vertical axis. A parametric Student's t test was used for the statistical analyses; ****p-value < 0.0001.

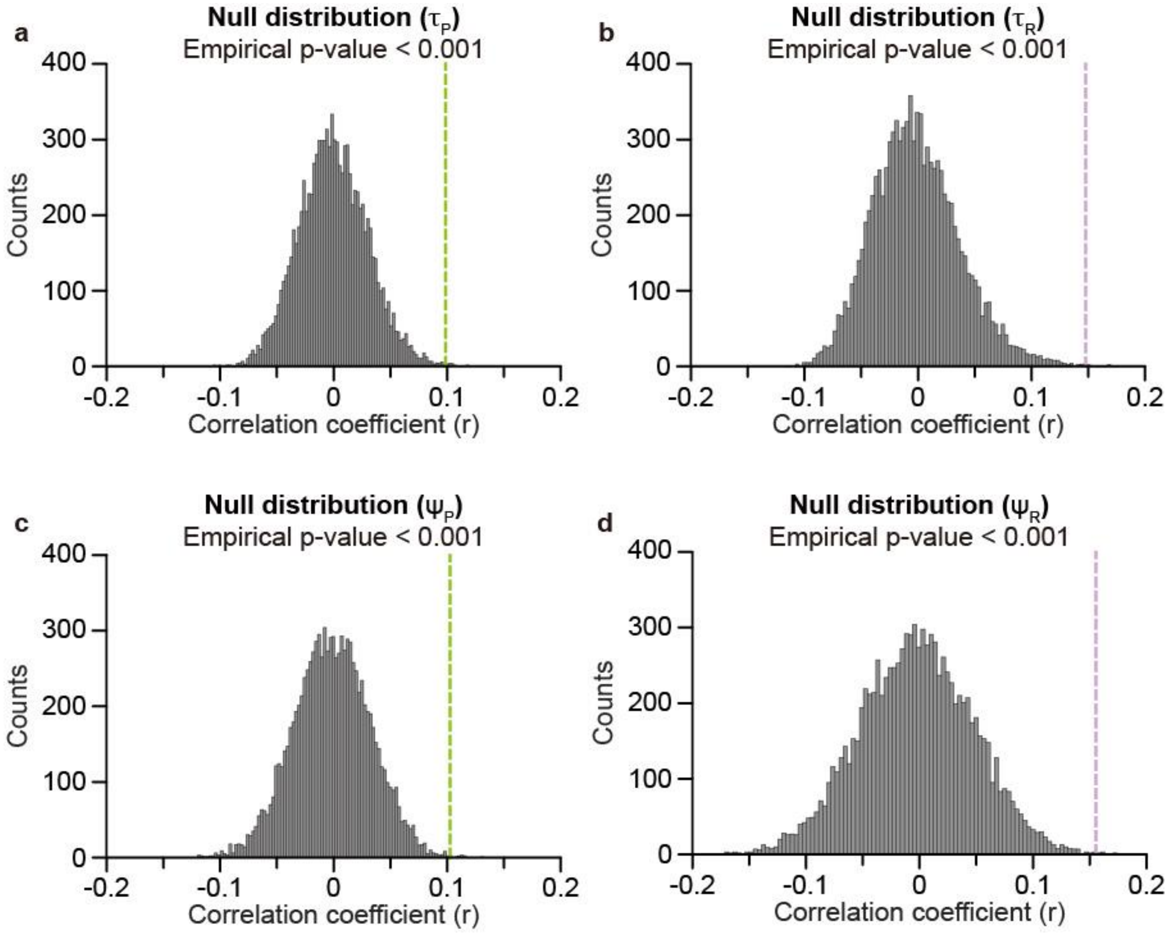

Figure S9. Defining the cut-off value for the significant SCC. a−d) Empirical null distribution of correlation coefficient (r) for inter-rotational duration at pause (τ_P) (a), inter-rotational duration at run (τ_R) (b), great circular angle change in endosomal rotation at pause (ψ_P) (c), and great circular angle change in endosomal rotation at run (ψ_R) (d). To determine the cut-off value for SCC, 10,000+ synthetic sequences of rotational angles were generated by random permutation of the original sequential data. The cut-offs for statistical significance were determined by using the value corresponding to a p-value of 0.001 in the empirical null distribution.

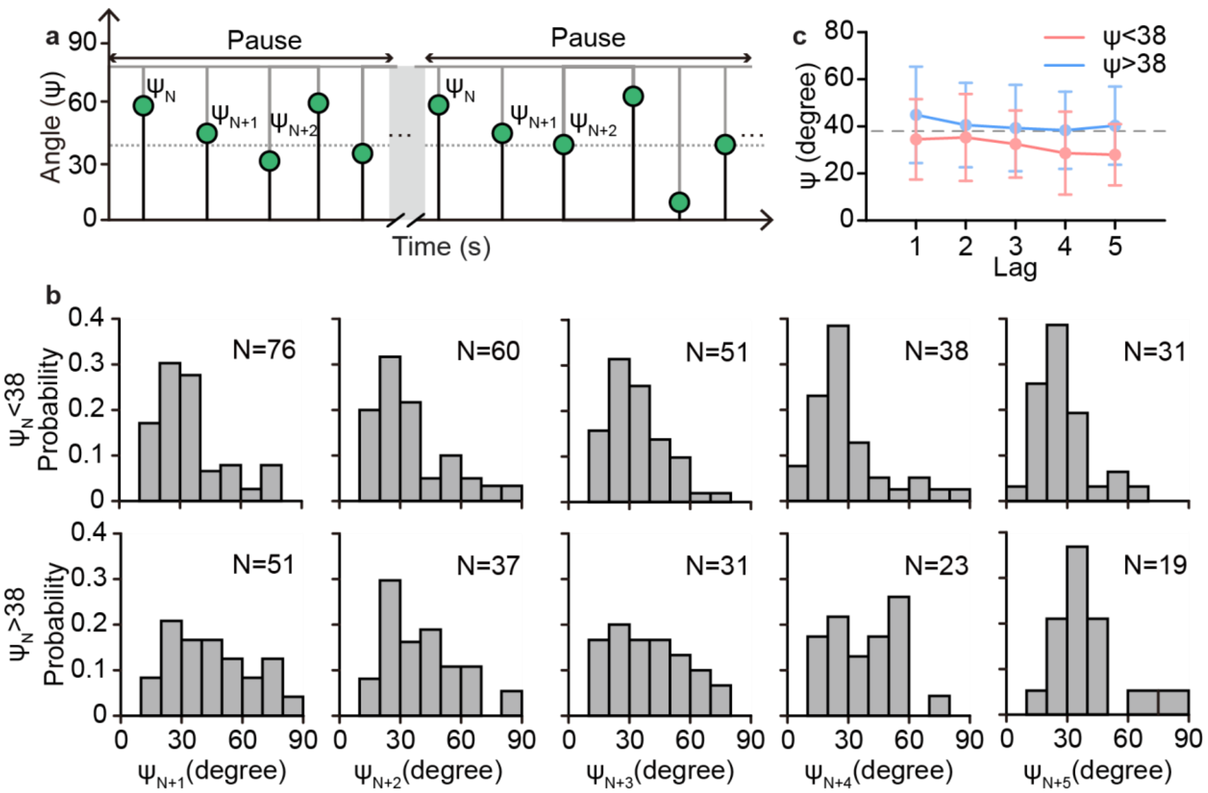

Figure S10. Time-dependent angle histogram at pause. Time-dependent angle distribution after large or small rotation in the pause state. a) Schematic illustrations explaining the time-dependent histogram as the following $\psi_{N+value}$ is distinguished. b) Time-dependent angle histogram of $\psi_{N+value}$, which was divided when $\psi_N > 38$ degrees (99% confidence interval of tremor distribution). c) Comparison of statistical values for $\psi_{N+value}$. Circles and error bars show the average and standard deviation of $\psi_{N+value}$, respectively. We observed that cases with initially large values tended to maintain large values, whereas cases with initially small values tended to sustain small values throughout the subsequent measurements.

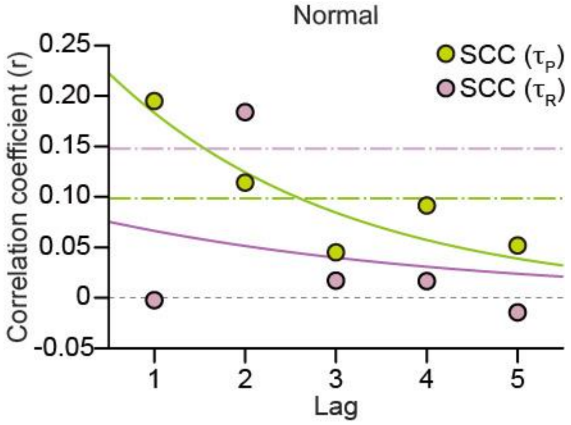

Figure S11. SCC of τ_P *and* τ_R. The τ_P exhibits a significant positive *r* for lag 2, whereas τ_R shows a negligible correlation. Statistical cut-offs were determined by calculating the top 0.1% value in the empirical null distribution of *r*, which was obtained by randomly permutating the order of original rotation sequences.

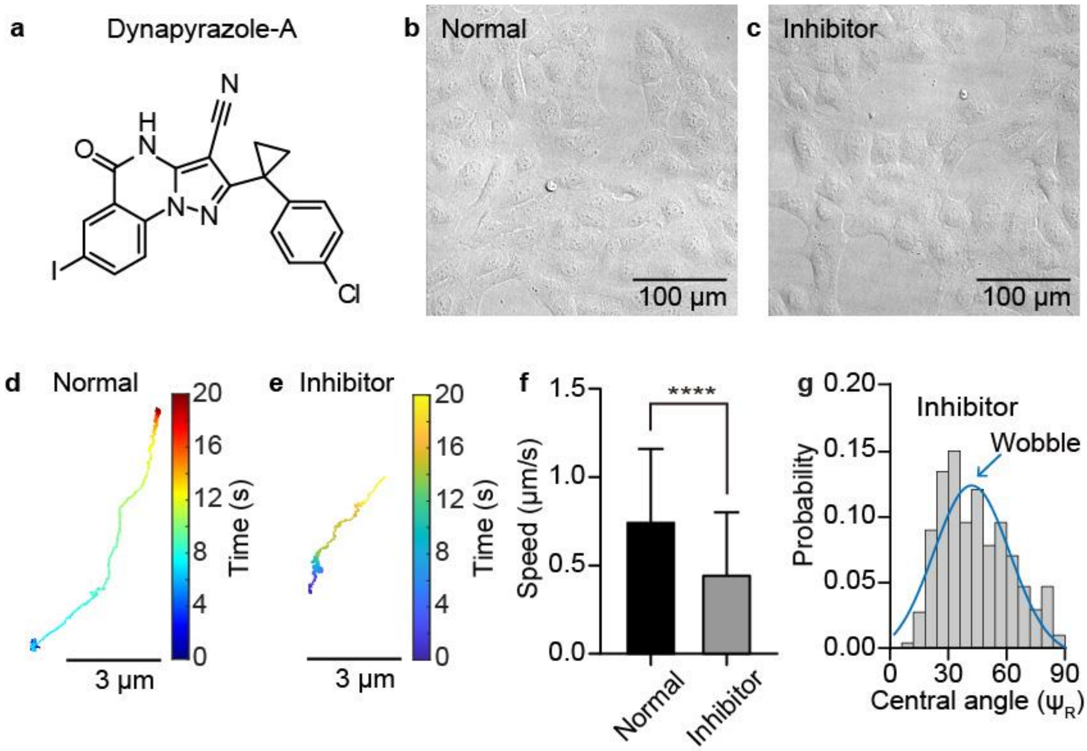

Figure S12. Inhibition of dynein-1 motor protein by dynapyrazole-A. a) Molecular structure of dynapyrazole-A. b, c) DIC images of living U2OS cells. There was no significant difference in the cellular morphology between the control (b) and inhibitor-treated (c) conditions. d, e) Observed endosomal trajectory under normal (d) or inhibitor-treated (e) conditions. f) Statistical analysis of flight speed under both conditions. Compared to the normal condition, the inhibitor decreased the speed of the flight. g) Probability histogram for ψ_P under inhibitor conditions (n = 512 for the rotational event at run). The graph was fitted with a Gaussian distribution. A parametric Student's t test was used for the statistical analyses; ****p-value < 0.0001.

Supporting Movie Legends

Movie S1. Process of pdf-FT acquisition from synthetic image sequences postulating the abrupt 3D rotation of an Au NR. Schematic illustration depicting the acquisition process of FT-pdf microscopy (left). The simulated DF images suggested the abrupt change in an Au NR under rotating linearly polarized light with a rotation rate of 360° s^{-1}. The intensity time trace was analyzed with the STFT algorithm, showing the few errors between the input parameter (φ, θ) and the numerically calculated value (right). The video is played in real time, with a 2 s interruption between the rotations of Au NR.

Movie S2. Time-lapse video of the trajectory and angular change in an endocytic vesicle in a living cell. DF microscopy video showing the Au NR inside the endosome. Images were captured under rotating linearly polarized light with a rotation rate of 360° s^{-1} (left). Trajectory and scattering intensity trace (middle). The red color indicates the pause, and the blue represents the run. Azimuthal (φ) and polar angle (θ) trace analyzed by the STFT algorithm (right). The video is played in real time.
